# Is Spelling an Important Factor in Examination?

**Published:** 1902-01

**Authors:** 


### IS SPELLING AN IMPORTANT FACTOR IN EXAMINATION?

Closely connected with the subject of the previous article is that recently made prominent at the meeting of the National Dental Association at Milwaukee.

Upon the programme of this body was a paper by Dr. John S. Marshall, on the "Organization of the Dental Corps of the United States Army." When this paper was read, it was received with evident marks of satisfaction by the majority, as it was a full statement of facts showing the results of this new work and the means taken to secure the greatest proficiency. The writer of this made but one criticism to this otherwise excellent paper, and this was to the following paragraph: "Candidates have presented themselves before our Board who were unable to write a sentence of twenty words without misspelling at least one-fourth of them. . . . Many could neither speak nor write without transgressing many of the rules of English grammar. We, however, are not alone in this, for the Army Medical Board now in session are having the same class of candidates come before them. I need not say that such men did not succeed in passing the examinations of the Army Dental Board, for they generally gave up the attempt after trying two or three subjects. The results of these examinations, it would seem to me, prove very conclusively that there is a great need of raising the standard of the entrance requirements of our dental colleges."

The hope was expressed that this paragraph would be omitted from the official report; but this has not been done, and it remains, as originally read, a reflection upon the intelligence and culture of the dental profession. Coming from this source, it will be accepted at home and abroad as a true statement of the mental training of the dental graduates of America. As it stands, it is a serious libel upon the educational institutions of this country; for, in the last analysis, the blame, if any exists, must be placed upon the shoulders of the educators of the land, both public and private, and not upon the professional schools that must perforce take the product as they find it from the high schools and colleges.

In the opinion of the writer, the standard enforced by the Army Board is, in the light of facts, an unjust one, and does not speak well for the broad intelligence of a body dealing with this important matter.

The spelling of English words has been from the inception of the language a trial to all classes; and it is a question whether there is a single individual, from childhood to old age, who is not obliged to refer frequently to the dictionary to be assured of correctness in spelling.

The methods devised by the older educators, and which were measurably successful, have seemingly been abandoned, for many of the present graduates of our high schools and universities seem to be deficient in ability to spell correctly some of the simplest words. That this will be increased is certain, for chirography is not as thoroughly taught as formerly. Orthography is fast becoming a lost art, and from present indications the present century will end with spelling left to proof-readers, printers, type-writers, and stenographers, and the ability to write a legible hand lost to the world.

In illustration of this, and confirmatory of its truth, the following quotations are taken from a recent article in a St. Louis journal giving interviews with prominent educators upon this subject.

"'The ability to spell well, it seems to me, is not worth what it costs,' said Chancellor Winfield S. Chaplin, of Washington University, yesterday afternoon.

"He was discussing the recent trouble in Northwestern University, Chicago, where several students are now diligently conning their spelling-books before their instructor will permit them to pursue their studies farther.

"'The only use one has for spelling,' continued the Chancellor, 'is when he writes. You do not spell out the words you use in conversation. You do not spell the words you hear in order to understand them. You do not stop to spell the words you read. And even when it comes to writing, one can express himself almost as well by bad spelling as by good.

"'This is not to be taken as an excuse for carelessness in orthography, and in Washington University we use every reasonable effort to make the students perfect in this branch. . . .

"'English is about the worst language in this respect. And some persons appear to be utterly incapable of learning to spell. One of my former pupils, whose orthography was especially bad, told me that he had learned to read by the word system, and had never been able to tell what letters composed the words he used. The habit of mind, once formed, he could not overcome, though he tried hard.

"'For myself, I used to rather pride myself on my spelling, but it is becoming a lost art with me. I use a stenographer for nearly everything I have to write, and when I do undertake to write a letter myself, I sometimes run across a very common word I am not sure about until I have written it out to see what it looks like.'

"Chicago schools have been criticised by Professor Clarke, of Northwestern University, because they pay little attention to spelling in the old-fashioned way. The 'word method,' he asserts, is responsible for the shortcomings of the young men who come to him for instruction in the higher English branches. By that method the pupil is taught to identify words as a whole, but never gets any definite idea of the parts of the word or its origin.

"Principal Bryan, of the High School, said, 'To spell well requires lifelong and continuous study. So many words are spelled without regard to their pronunciation that it is a severe tax on the memory to bear them all in mind at once.'"

It is in the experience of all educators that the faculty of spelling is not at all natural to the individual, for, as Superintendent Soldan declared, "Spelling is not a natural gift with anybody." It cannot be compared with the linguistic and mathematical faculties, these being usually an inheritance. A distinguished English writer acknowledged he was never able to accomplish the multiplication table. In truth, there is an idiotic side to the mentality of every human being, or, to adopt the teaching of Dr. Talbot, we are, in some respects, all degenerates.

